# Phase Diagram of Water Confined by Graphene

**DOI:** 10.1038/s41598-018-24358-3

**Published:** 2018-04-18

**Authors:** Zhenghan Gao, Nicolas Giovambattista, Ozgur Sahin

**Affiliations:** 1 Department of Physics, Columbia University, New York City, NY 10027 USA; 2 Department of Biological Sciences, Columbia University, New York City, NY 10027 USA; 3 Departments of Physics, Brooklyn College of the City University of New York, Brooklyn, NY 11210 USA; 4 PhD Programs in Physics and Chemistry, The Graduate Center of the City University of New York, New York City, NY 10016 USA

## Abstract

The behavior of water confined at the nanoscale plays a fundamental role in biological processes and technological applications, including protein folding, translocation of water across membranes, and filtration and desalination. Remarkably, nanoscale confinement drastically alters the properties of water. Using molecular dynamics simulations, we determine the phase diagram of water confined by graphene sheets in slab geometry, at *T* = 300 K and for a wide range of pressures. We find that, depending on the confining dimension D and density *σ*, water can exist in liquid and vapor phases, or crystallize into monolayer and bilayer square ices, as observed in experiments. Interestingly, depending on D and *σ*, the crystal-liquid transformation can be a first-order phase transition, or smooth, reminiscent of a supercritical liquid-gas transformation. We also focus on the limit of stability of the liquid relative to the vapor and obtain the cavitation pressure perpendicular to the graphene sheets. Perpendicular cavitation pressure varies non-monotonically with increasing D and exhibits a maximum at *D* ≈ 0.90 nm (equivalent to three water layers). The effect of nanoconfinement on the cavitation pressure can have an impact on water transport in technological and biological systems. Our study emphasizes the rich and apparently unpredictable behavior of nanoconfined water, which is complex even for graphene.

## Introduction

Water is ubiquitous on Earth and plays a central role in numerous scientific and technological applications. This is particularly true for the case of water confined at the nanoscale, which has received a great deal of attention in both experimental and numerical studies in diverse scientific disciplines, including biology^1–6^, engineering^7–13^, chemistry^14,15^, and material science^16,17^. The behavior of water confined at the nanoscale can be remarkably different from the well-known behavior of *bulk* water^18^. For example, while at normal pressure water crystallizes into hexagonal ice, nanoconfined water may crystallize into a plethora of novel ices, never seen in bulk water at low/high pressure^19–21^. In addition, nanoconfined water can exhibit substantially higher phase transition temperatures than bulk water^22–24^. Nanoconfinement not only affects water’s thermodynamic properties; dynamical properties are usually affected as well. For example, the translocation and permeation of water within 1D and 2D nano channels formed by carbon nanotubes and graphene sheets exhibit significant enhancements relative to the bulk^25,26^. In other cases, water’s viscosity, and the associated shear forces, can increase by orders of magnitude relative to bulk water^27–29^. Understanding the behavior of water under extreme confinement could provide insights into various scientific problems in surface chemistry, and facilitate the development of novel applications that benefit from water’s anomalous behavior induced by nanoscale confinement.

Numerous theoretical and computational studies, including density functional theory and molecular dynamics (MD) simulation, have provided considerable insights into the structure, thermodynamic, and dynamical properties of water confined within nanocapillaries^21,23,30–33^. Common confining model surfaces include detailed realistic surfaces, such as carbon nanotubes (CNTs)^11,21,34–36^, graphene sheets^23,31,32,37,38^, *SiO*_2_^33,39^, and *MoS*_2_ nanopores^40,41^, which have potential applications in water desalination and purification^41,42^, as well as model surfaces, such as unstructured smooth confining walls^32,43,44^. These and other studies show that the unique properties of nanoconfined water depend strongly on the confining geometry and dimensions^36^, and characteristics of the confining surfaces, such as chemistry^45^, structure^33^, and curvature^46^. Unfortunately, at present, water’s behavior at the nanoscale is rather unpredictable and results from one study, based on a specific confined system, are not necessarily transferable to other confined systems.

Confining geometries based on graphene are of particular interest due to graphene’s unusual properties, including high strength^47^, optical transparency^48^, and high electrical conductivity^49^. In this regard, we note that water’s unusual behavior in contact with or confined by graphene can largely affect graphene’s properties. In the context of confined water, graphene is unique because of its atomically smooth and uniform structure; graphene is perhaps one of the simplest confining surfaces that one could use to study confined water. Numerous experimental and computer simulations studies of water confined within carbon nanotubes are available^11,21,24,50,51^. These studies show that confinement can induce vaporization of water at unexpected low temperatures, or induce ice formation into novel structures, such as tubular square and hexagonal ices^18,21^. Similarly, the phase behavior of water confined by graphene sheets is very rich. Recent transmission electron microscopy studies at room temperature and computer simulations show that water confined by parallel graphene sheets can crystallize into novel structures such as monolayer and bilayer square ices^23,31,52^.

At present, a full exploration of the phase diagram of water confined by graphene sheets is not available. A first principle computational study shows a complex ice phase diagram that includes a monolayer ice^31^. However, this study was conducted only at *T* = 0 K. Recent MD simulations of water confined by graphene sheets explored the phase diagram of water at 100 K < *T* < 400 K and pressures in the range 0.1–5 GPa^53^. This study finds the formation of monolayer square ice and bilayer triangular AA stacking ice, depending on the pressure. However, this work is limited to the case of graphene sheets separation *D* = 0.9 nm. Another recent study included the effects of varying *D* at constant temperature, though the range was narrow (0.65 nm < *D* < 0.75 nm) and lateral pressures were high (>500 MPa)^54^.

In this work, in order to improve our understanding of nanoconfined water, we study systematically the phase diagram of water confined by two parallel graphene sheets. A complete phase diagram of water under these conditions could be characterized in terms of the separation between sheets D, temperature T, number of water molecules N, and walls surface area A. A complete 4D phase diagram is very complex and difficult to analyze and hence, we limit ourselves to the case *T* = 300 K. We explore graphene sheets separations *D* = 0.6–1.5 nm and a wide range of densities that encompass the liquid and vapor states as well as crystallization (into monolayer and bilayer square ices). We pay particular attention to the behavior of nanoconfined water under tension, which has been mostly overlooked. Due to the strong surface tension of water, *bulk* water can withstand very negative pressures (approximately −100 MPa at *T* = 300 K), in agreement with estimations based on Classical Nucleation Theory (CNT)^55,56^. Interestingly, we find that the cavitation pressure of water confined by graphene is highly sensitive to the confining dimension and varies non-linearly with D, covering a range of ~500 MPa (0.85 < *D* < 1.35 nm). We confirm that the phase diagram we obtain is qualitatively unchanged if we alter water carbon interactions (corresponding to water contact angles in the range 90–110°) and confirm that our phase diagram is independent of the methodology employed, as expected.

## Results

**Figure 1:**
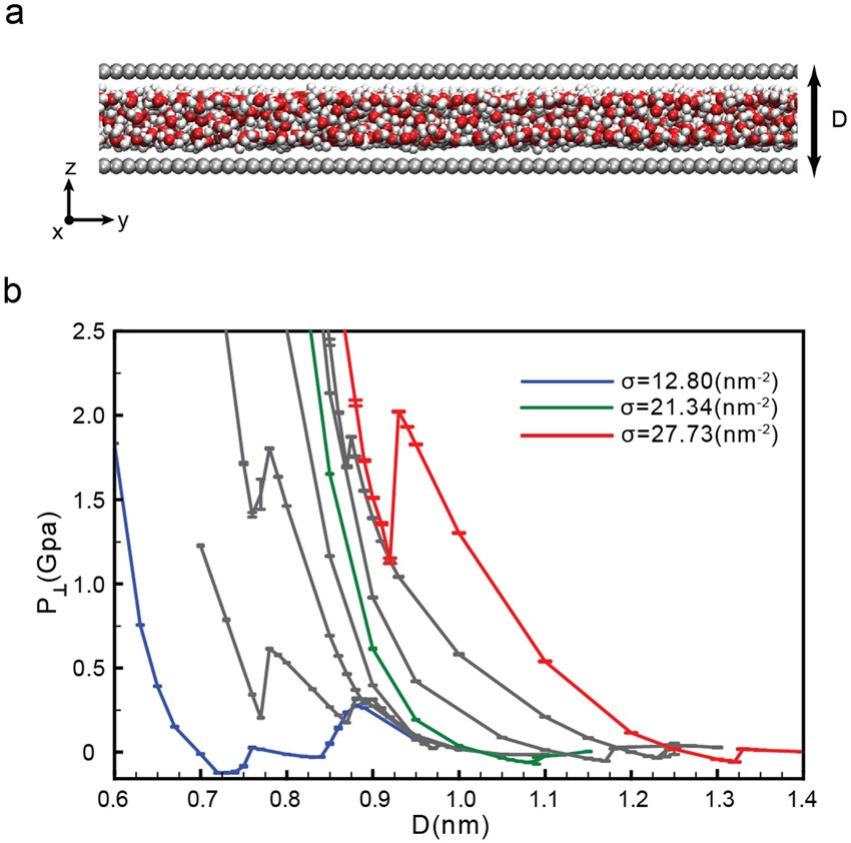
(**a**) Snapshot of the system studied in this work where water is confined by two parallel graphene sheets. Periodic boundary conditions are applied along the x and y directions and hence, the graphene sheets are effectively infinite. (**b**) Pressure perpendicular to the graphene sheets as a function of D for selected surface densities *σ* = *N*/*A*. *σ* increases from 12.80 nm^−2^ (left curve) to 27.73 nm^−2^ (right curve) in increments of 2.13 nm^−2^. At *σ* < 17.06 nm^−2^, water exhibits two phase transitions, indicated by the sudden increase in *P*_⊥_(*D*). At small D, water evolves from a monolayer square ice to the liquid state; at large D, water exhibits a liquid to vapor phase transition. At 19.19 < *σ* < 23.45 nm^−2^, water shows only a liquid to vapor phase transition at large values of D. At *σ* > 25.60 nm^−2^, water exhibits a bilayer ice to liquid transition at small D, and a liquid to vapor phase transition at large D.

Our results are based on the system shown in Fig. 1a where water is confined by two “infinite” parallel graphene sheets, separated by a distance D (see Methods). We perform molecular dynamics (MD) simulations at constant number of molecules N (at *T* = 300 K), and vary the walls separation from *D* ≈ 0.6 nm (corresponding to a water monolayer) up to *D* ≈ 1.4 nm. For a given N, we calculate the pressure perpendicular to the walls as a function of the walls separation, *P*_⊥_(*D*). We note that knowledge of *P*_⊥_(*D*) is sufficient to identify phase transitions between the different phases accessible to the system (at a given N and T). Specifically, as shown in the Supplementary Information (SI), at constant A, *P*_⊥_(*D*) must be a monotonic decaying function of D for the system to be stable, and the system experiences a first-order phase transition if (∂*P*_⊥_/∂*D*)_*N*,*A*,*T*_ > 0^57,58^.

We divide the results into three parts. In the first part, we describe in detail the different phase transitions observed in water confined by graphene sheets. In the second part, the complete phase diagram of water confined by graphene sheets is presented. In the last part, we test the consistency of our phase diagram with independent simulations of water confined by graphene sheets in contact with an external water reservoir. We conclude with a brief summary of the results presented in this work.

### Phase Transitions in Water Confined by Graphene Sheets

We perform MD simulations for 3000 ≤ *N* ≤ 6500 depending on D, corresponding to surface number densities *σ* ≡ *N*/*A* in the range 12.80 ≤ *σ* ≤ 27.74 nm^−2^ (here, *A* = 15.386 × 15.228 nm^2^ is the graphene sheets surface area). Although the behavior of *P*_⊥_(*D*) varies considerably with *σ*, we find that the qualitative behavior of *P*_⊥_(*D*), and the associated phase transitions, fall within one of the following three scenarios (see Fig. 1b).

**Figure 2:**
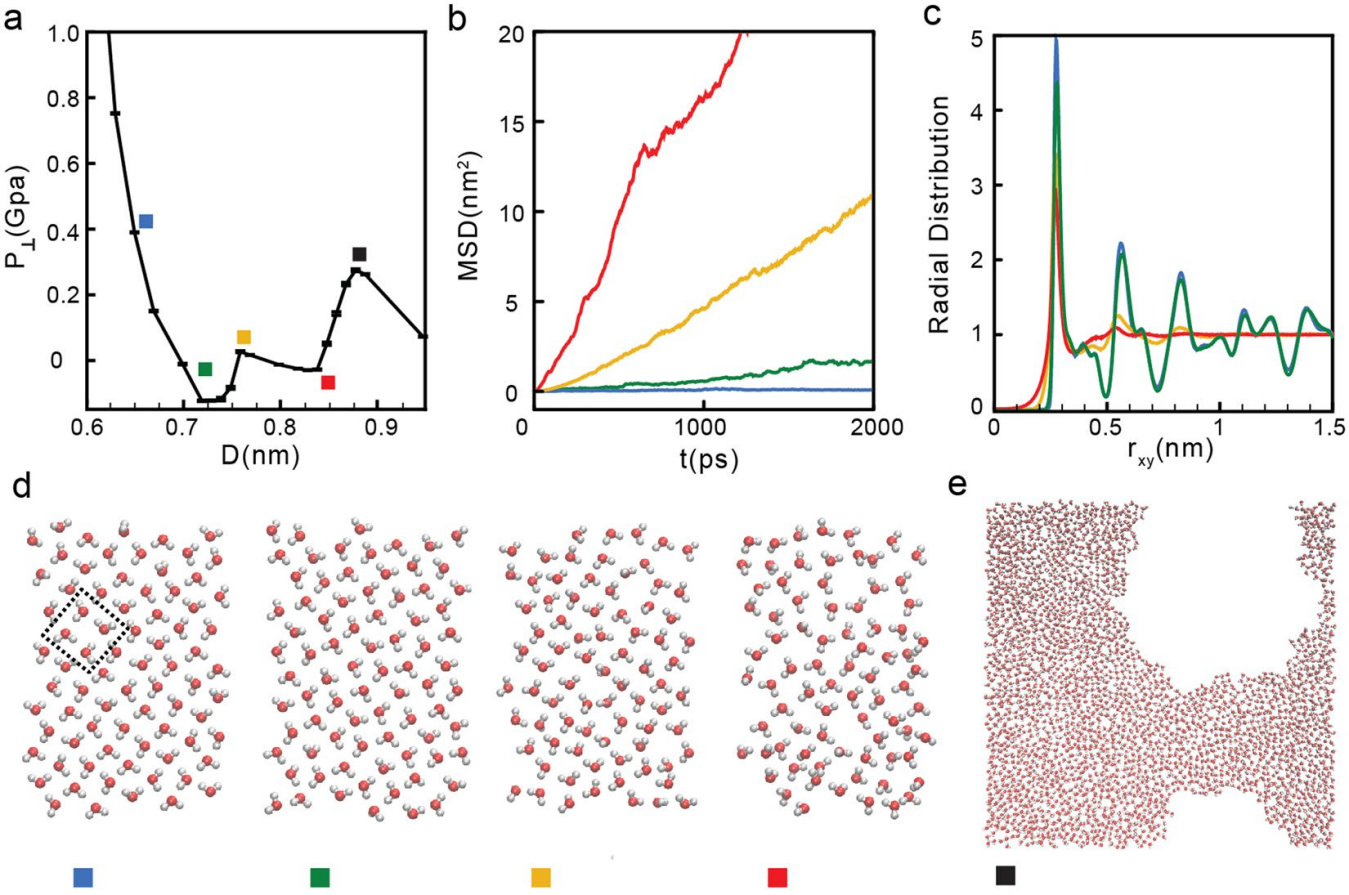
(**a**) Pressure perpendicular to the graphene sheets as a function of D at *σ* = 12.80 nm^−2^ (blue curve in Fig. 1b). Water crystallizes into a monolayer ice at *D* < 0.74 nm (green square), remains in the liquid state at 0.77 < *D* < 0.83 nm (yellow and red squares) and is in the vapor state at *D* > 0.83 nm (black square). (**b**) Mean-square displacement parallel to the walls for water molecules confined at *D* = 0.83 nm (red), 0.77 nm (orange), 0.74 nm (green), and 0.65 nm (blue), corresponding to the squares in (a). (**c**) Water OO radial distribution function projected on the xy-plane. (**d**) Snapshots taken along the z-axis for the graphene sheets separations indicated in (a) (squares). For all values of D, water molecules arrange into a single layer parallel to the sheets. (**e**) Snapshot of confined water at *D* = 0.87 nm (black square) where liquid water is unstable relative to the vapor.
  The phase behavior of the system is determined based on the mean square displacement (MSD) of the molecules *parallel* to the walls, *MSD*(*t*)^60^; the oxygen-oxygen radial distribution function (RDF) *parallel* to the walls, *g*_*OO*_(*r*)^32,60^ and visual inspection of snapshots taken at different separations. In addition, to discriminate among multilayer structures, we also calculate the transverse density profile along the *z*-direction, *ρ*_*slab*_(*z*)^60^. Figure 2b and c show the *MSD*(*t*) and *g*_*OO*_(*r*) for *σ* = 12.80 nm^−2^ and for selected walls separations (indicated in Fig. 2a with squares). At *D* < 0.74 nm, the system is in the solid state. Accordingly, as shown in Fig. 2b (blue and green lines), the *MSD*(*t*) reaches a plateau at long times indicating that molecular translational motion is absent for long times^21,32,61–63^. Snapshots of the systems at *D* < 0.74 nm clearly show that water molecules arrange in a monolayer square lattice (see snapshots with blue and green squares in Fig. 2d). The ice formation is also evident from the corresponding *g*_*OO*_(*r*) (blue and green lines in Fig. 2c). At these small values of D, *g*_*OO*_(*r*) exhibits pronounced maxima and minima at all values of r_xy_, indicative of long range order^60^.
  At walls separations 0.83 < *D* < 0.87 nm (yellow and red squares in Fig. 2a), the system is in the liquid state. This is consistent with the *MSD*(*t*) at these separations (yellow and red lines in Fig. 2b) which increases monotonically with increasing time. In addition, snapshots of the system at these separations (Fig. 2d, yellow and red squares) indicate that molecules arrange in an amorphous monolayer structure. The absence of long range order, characteristic of the liquid state, is confirmed by *g*_*OO*_(*r*) which is ~1 for *r*_xy_ > 0.7 nm (Fig. 2c, yellow and red lines).
  A comparison of the snapshots in Fig. 2d for *D* = 0.77 nm and *D* = 0.83 nm (yellow and red squares), indicates that as D increases, the liquid becomes less dense and the molecules distribution is less uniform. This suggests that as *D* → 0.83 nm (red square), the propensity to observe small cavities is growing. Indeed, at *D* ≈ 0.83 nm, the systems exhibits a liquid-to-vapor phase transition and, at approximately *D* > 0.83 nm, we observe cavitation in the system (Fig. 2e).
(ii) *Intermediate densities*: 19.19 ≤ *σ* ≤ 23.45 nm^−2^. As shown in Fig. 1b, at these densities, *P*_⊥_(*D*) exhibits only one region of instability [(∂*P*_⊥_/∂*D*)_*N*,A,*T*_ > 0]. At small walls separations, water is liquid while for large walls separations, water is in the vapor state^64^. As an example, we discuss the results for *σ* = 21.34 nm^−2^. At this density, the liquid-vapor phase transition occurs at *D* ≈ 1.09 nm; see Fig. 3a.

(i) *Low densities*: 12.80 ≤ *σ* ≤ 17.06 *nm*^−2^. For low water contents, *P*_⊥_(*D*) exhibits two regions of instability [(∂*P*_⊥_/∂*D*)_*N*,A,*T*_ > 0] and hence, the system experiences two first-order phase transitions^59^; see Fig. 1b. For example, at the lowest density we can investigate, *σ* = 12.80 nm^−2^, *P*_⊥_(*D*) shows two unstable regions, one at 0.74 ≤ *D* ≤ 0.77 nm and the other at 0.83 ≤ *D* ≤ 0.87 nm; see Fig. 2a. As we show below, water crystallizes rapidly into a monolayer square ice at very small walls separations (*D* < 0.74 nm nm in Fig. 2a) and, upon increasing D, the monolayer ice melts into a monolayer liquid (0.77 ≤ *D* ≤ 0.83 nm in Fig. 2a)^23^. At larger walls separations (*D* > 0.83 nm in Fig. 2a) we observe cavitation indicating that the liquid becomes unstable relative to the vapor phase. The sequence of transformations ‘monolayer ice → liquid → vapor’ with increasing D is found at all densities 12.80 ≤ *σ* ≤ 17.06 nm^−2^.

**Figure 3:**
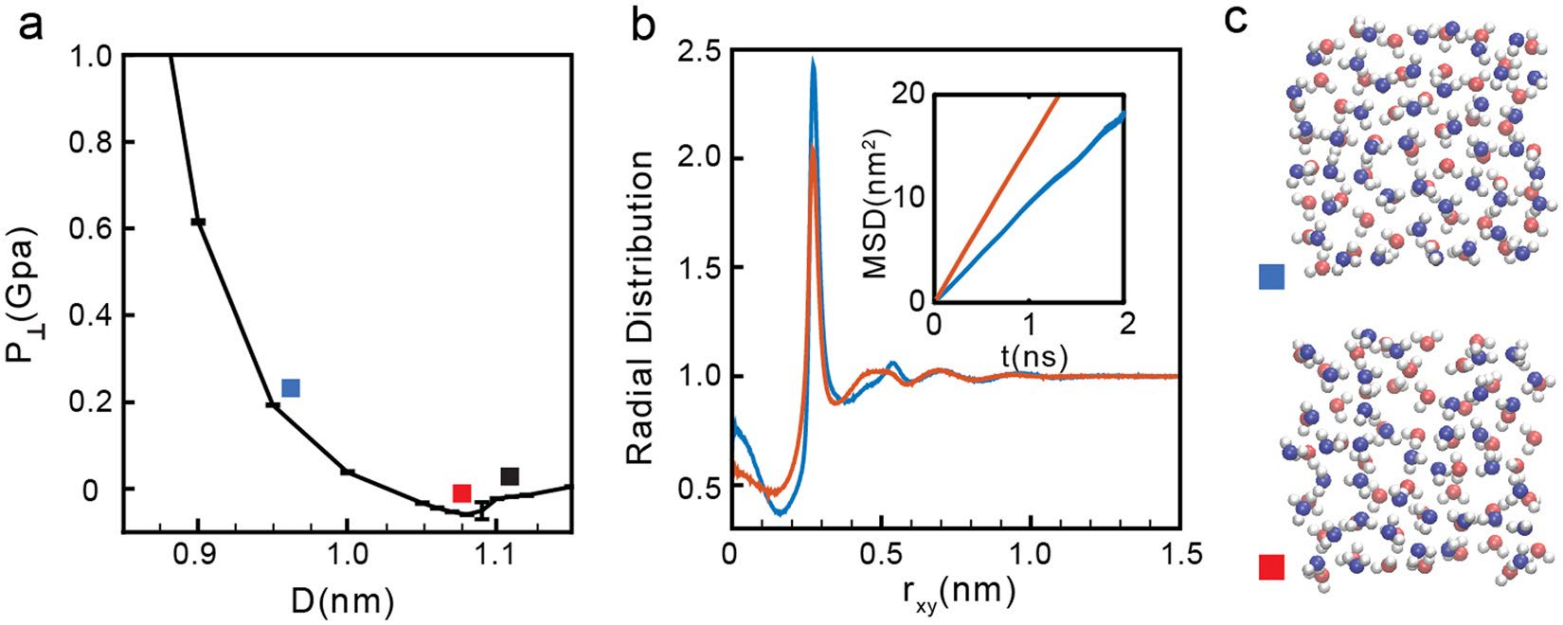
(**a**) Pressure perpendicular to the graphene sheets as a function of D at *σ* = 21.34 nm^−2^ (green curve in Fig. 1b). Water is in the liquid state at *D* < 1.09 nm (red square) and transforms to the vapor state at *D* > 1.09 nm. (**b**) OO radial distribution function and water MSD parallel to graphene sheets for *D* = 0.95 nm (blue square) and 1.09 nm (red square). At these graphene sheets separations, water molecules arrange into two layers. (**c**) Snapshots at *D* = 0.95 nm (blue square) and *D* = 1.09 nm (red square) showing molecules in red and blue that belong to different layers. At *D* > 0.95 nm (see, e.g., black square), water exhibits cavitation (see e.g. Figure 2e).
  The *MSD*(*t*) and *g*_*OO*_(*r*) for the case *σ* = 21.34 nm^−2^ are shown in Fig. 3b for selected walls separations (indicated by blue and red squares in Fig. 3a). The behavior of *MSD*(*t*) and *g*_*OO*_(*r*) at these walls separations are consistent with the results obtained at low densities in the liquid state. Specifically, the *MSD*(*t*) are increasing functions of time (inset of Fig. 3b), indicating that water molecules are able to diffuse. In addition, as expected for a liquid, *g*_*OO*_(*r*) exhibits no oscillations for large r, indicating that the system is indeed amorphous. Analysis of *ρ*_*slab*_(*z*) indicate that water molecules arrange into two layers parallel to the wall. Snapshots of the bilayer liquid are included in Fig. 3c.
(iii) *High densities*: *σ* ≥ 25.60 nm^−2^. At high densities, *P*_⊥_(*D*) exhibits two regions of instability (see Fig. 1b) and hence, the system experiences two first-order phase transitions. Similarly to the behavior found at low densities, water crystallizes at low D, it remains in the liquid state for intermediate values of D, and it is in the vapor state at large walls separations. However, while water crystallizes into a monolayer square ice at *low* densities, at *high* densities, water crystallizes into a bilayer square ice (see below).
  As an example, we discuss the phase behavior of water at *σ* = 27.73 nm^−2^, see Fig. 4a. At this density, the ice-liquid and liquid-vapor phase transitions occur at approximately *D* = 0.92 nm and *D* = 1.32 nm, respectively. The *MSD*(*t*) and *g*_*OO*_(*r*) for selected states are included in Fig. 4b. As found at low and intermediate densities, we find that while the system is in the liquid state, 0.93 < *D* < 1.32 nm, the *MSD*(*t*) increases monotonically with time (see red and yellow lines in the inset of Fig. 4b and squares in Fig. 4a), as expected. In addition, the RDF at these separations (red and yellow lines in Fig. 4b) is constant for approximately *r*_xy_ > 1.00 nm, meaning that the system is amorphous. At the present density, however, the liquid structure evolves continuously with increasing D. Specifically, at the lowest value of D accessible to the liquid state, *D* = 0.93 nm (yellow square of Fig. 4a), water molecules form two layers. This is shown in Fig. 4c, where the density profile (yellow line) exhibits two pronounced maxima at *z* = ±0.15 nm. Instead, at the largest walls separation accessible to the liquid, *D* = 1.32 nm, water molecules form three layers (see red line in Fig. 4c). This bilayer liquid-to-trilayer liquid transformation is smooth. We note that as the walls separation is further increased, the trilayer liquid becomes unstable relative to the vapor phase. For *σ* = 27.73 nm^−2^, the trilayer liquid-vapor phase transition occurs at *D* = 1.32 nm. Accordingly, snapshots taken *D* > 1.32 nm exhibit cavitation.

**Figure 4:**
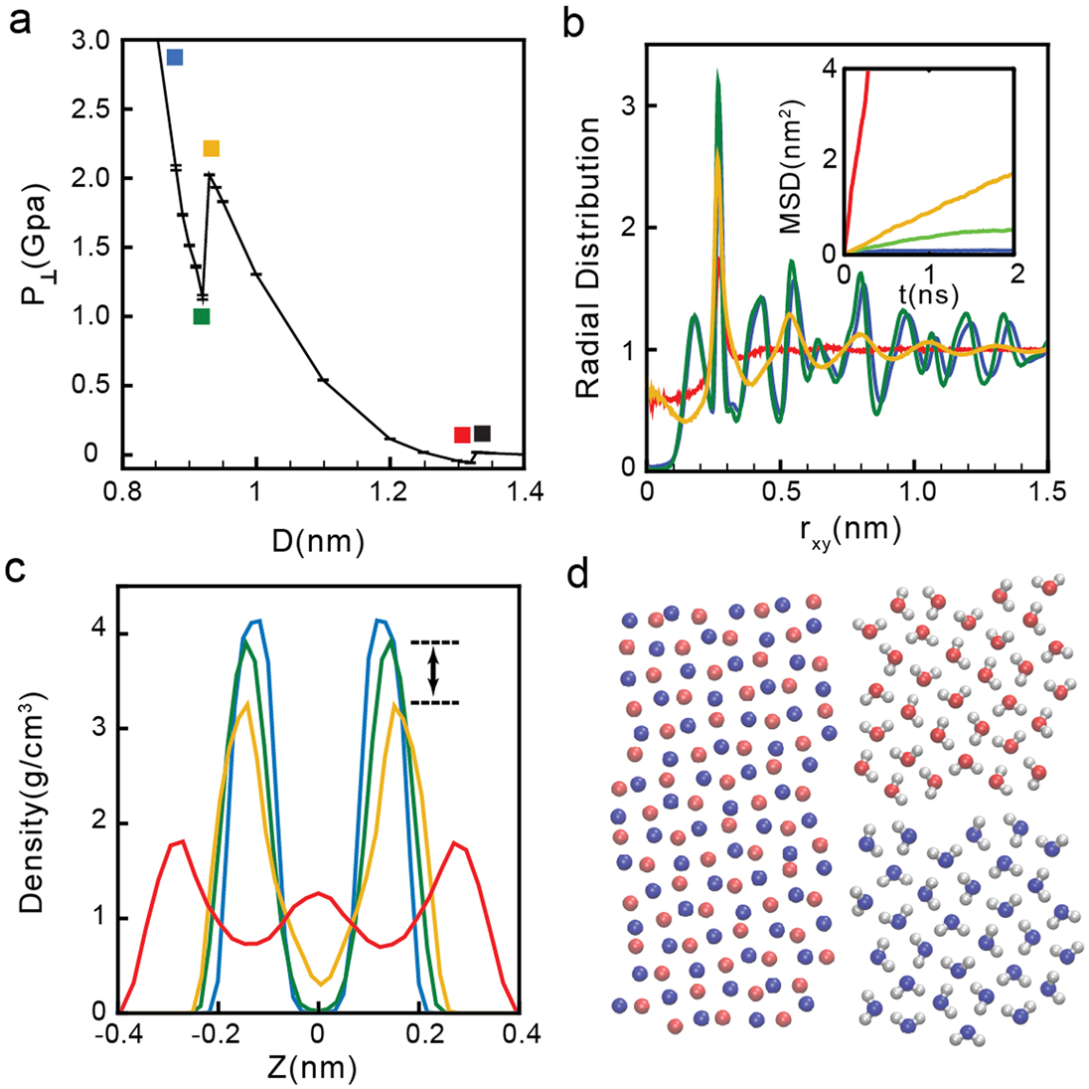
(**a**) Pressure perpendicular to the graphene sheets as a function of D at *σ* = 27.73 nm^−2^ (red curve in Fig. 1b). Water crystallizes into a bilayer ice at *D* < 0.92 nm (green square), remains in the liquid state at 0.93 < *D* < 1.32 nm (yellow and red squares) and is in the vapor state at *D* > 1.32 nm (black square). (**b**) OO radial distribution function and MSD (inset) parallel to the walls for water molecules confined at *D* = 1.32 nm (red), 0.93 nm (yellow), 0.92 nm (green), and 0.85 nm (blue), corresponding to the squares shown in (a). (**c**) Density profiles for the same values of D [lines are color-coded as in (b)]. (**d**) Snapshot of the system in the bilayer ice with molecules in blue and red molecules belonging to different layers.
  The bilayer ice at *σ* = 27.73 nm^−2^ forms at *D* < 0.92 nm; see Fig. 4a. At these walls separations, the *MSD*(*t*) becomes constant for long times (see blue and green lines in the inset of Fig. 4b) and the *g*_*OO*_(*r*) exhibits pronounced maxima and minima (see blue and green lines in Fig. 4b), which indicates that there is long range order in the water film. That the crystal is bilayer is indicated by the *ρ*_*slab*_(*z*) shown in Fig. 4c (blue and green lines).
  Snapshot of the water molecules for the case *D* = 0.92 nm (green square in Fig. 4a) is included in Fig. 4d. Molecules are colored red and blue depending on the monolayer they belong to. It follows from Fig. 4d that the crystal structure consist of two monolayers of square ice and that each of these monolayers is reminiscent of the monolayer square ice shown in Fig. 2d found at low densities. Interestingly, the two monolayers of square ice are out-of-registry (AB stacking) and they are *not* connected by hydrogen bonds (HBs)^23,65^. This is rather unusual since most ices in bulk and confined water are characterized by a continuous hydrogen-bond network. The bilayer ice in Fig. 4c is composed of two HB networks. Indeed, water molecules within a single ice monolayer have both OH covalent bonds oriented parallel to the walls. This molecular orientation allows water molecules to form approximately four HBs with other water molecules within the same monolayer. We note that the bilayer ice structure we obtain is not in agreement with the square bilayer ice reported in experiments but is consistent with previous simulation results^23^. Specifically, MD simulations showed AB stacking instead of AA stacking.

### Phase Diagram for Water confined by Graphene Sheets (*T* = 300 K)

**Figure 5:**
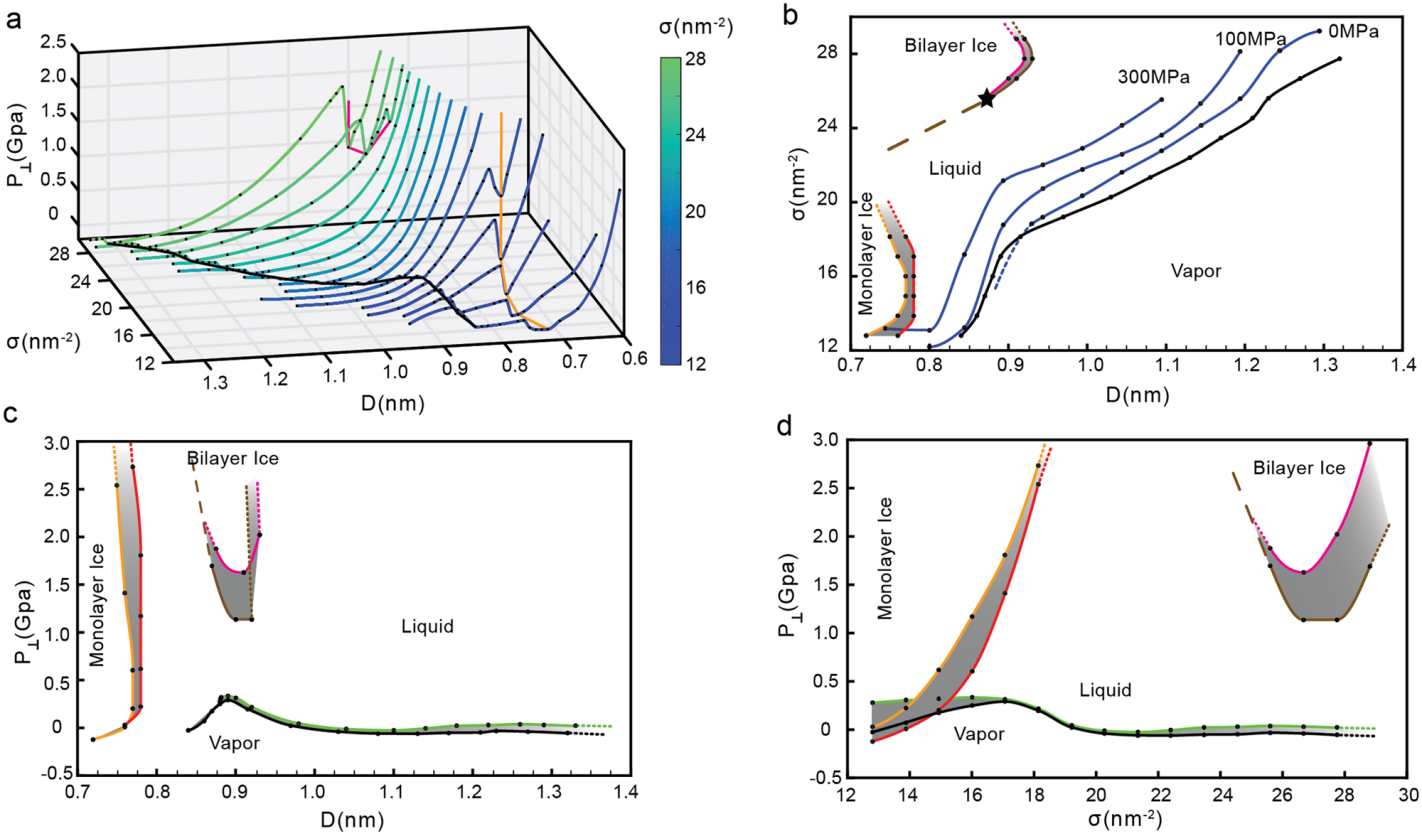
(**a**) Pressure perpendicular to the graphene sheets as a function of D and for all surface densities studied (including the values of sigma shown in Fig. 1b). (**b**)–(**d**) Phase diagrams of water confined by graphene sheets obtained by projecting the *P*_⊥_ − *D* − *σ* surface in (a) onto the *σ* − *D*, *P*_⊥_ − *D* and *P*_⊥_ − *σ* planes. The grey regions between the orange and red (brown and purple) lines indicates the instability region associated to the monolayer ice-liquid (bilayer ice-liquid) phase transitions. The black line is the limit of stability of the liquid relative to the vapor and corresponds to the cavitation pressure, ${P}_{\perp }^{cav}$. In (**b**), the star indicates the value of σ above which the bilayer ice-liquid transformation is a first order phase transition; below this density, the transformation is smooth (see text).

The phase behavior of water confined between parallel graphene sheets at *T* = 300 K is summarized in Fig. 5a. Figure 5a includes the *P*_⊥_(*D*) curves obtained for all the values of *σ* studied, including the *P*_⊥_(*D*) curves shown in Fig. 1b. The projections of the *P*_⊥_ − *D* − *σ* surface onto the *σ* − *D*, *P*_⊥_ − *D*, and *P*_⊥_ − *σ* planes are shown, respectively, in Fig. 5b–d. These 2D projections are three phase diagrams that characterize the behavior of water confined by graphene sheets at *T* = 300 K. Of particular relevance is the *σ* − *D* phase diagram of Fig. 5b. This is because, for a given point (*D*, *σ*) in Fig. 5a, *P*_⊥_ is univocally defined [i.e., *P*_⊥_ is a well-defined function of (*D*, *σ*)] and, hence, only one phase state for water can be identified. Instead, for a given (*P*_⊥_, *D*) point in Fig. 5a, there *may* be more than one value for *σ* that one can associate to water and hence, one or more phase states (stable/metastable) accessible to water. Similarly, for a given (*P*_⊥_, *σ*) point in Fig. 5a, there may be more than one value for D, corresponding to one or more phase states accessible to water. Below, we describe the phase diagrams of Fig. 5b–d in detail.

The four phases of water confined between graphene sheets, found at the studied values of D and *σ*, are indicated in Fig. 5b. The black line represents the limit of stability, or spinodal line, of the liquid relative to the vapor. For a given value of *σ*, this line is defined by the minimum of the corresponding *P*_⊥_(*D*) curve shown in Fig. 5a located at large values of D; for example, for *σ* = 27.73 nm^−2^, the minimum of *P*_⊥_(*D*) at large D is located at *D* = 1.35 nm; see Fig. 4a. The minima of the *P*_⊥_(*D*) curves associated to the liquid-to-vapor spinodal line are indicated by the black line in Fig. 5a.

The boundaries between the monolayer ice and the liquid are shown in Fig. 5b by orange and red lines. The orange line at small values of D represent the monolayer ice-to-liquid spinodal line while the red line at larger values of D represents the liquid-to-monolayer ice spinodal line. The grey region between these two spinodal lines represent the region of instability where the liquid and the monolayer ice coexist. At a given *σ*, these spinodal lines are defined by the corresponding minimum and maximum of *P*_⊥_(*D*). For example, at *σ* = 12.80 nm^−2^, Fig. 2a shows that the minimum of *P*_⊥_(*D*) at small D occur at *D* ≈ 0.72–0.74 nm; this value defines the location of the monolayer ice-to-liquid spinodal (orange) line in Fig. 5b. Similarly, the maximum of *P*_⊥_(*D*) for *σ* = 12.80 nm^−2^ (Fig. 2a) occurs at *D* = 0.76 nm and defines the liquid-to-monolayer ice (red) spinodal in Fig. 5b. The grey region in Fig. 5b corresponds to the range of D where (∂*P*_⊥_/∂*D*)_*N,A,T*_ > 0, during the monolayer ice-to-liquid first-order phase transition.

The boundaries between the bilayer ice and the liquid are shown in Fig. 5b by magenta and brown lines. The magenta line at small values of D represent the bilayer ice-to-liquid spinodal line while the brown line at larger values of D represents the liquid-to-bilayer ice spinodal line. The grey region between these two spinodal lines represents the region of instability where the liquid and the bilayer ice coexist. As for the case of the *mono*layer ice-liquid (previous paragraph), the magenta and brown spinodal lines in Fig. 5b are defined by the corresponding minima and maxima in the *P*_⊥_(*D*) lines of Fig. 5a. For comparison, the magenta line shown in Fig. 5a indicates the minima in *P*_⊥_(*D*) that define the bilayer ice-to-liquid spinodal line (corresponding to the magenta line in Fig. 5b).

An uncommon feature of confined water regarding the liquid-bilayer ice transition follows from Fig. 5b. Specifically, our MD simulations show that at *σ* ≥ 25.60 nm^−2^, the liquid-bilayer ice transformation is a first-order phase transition, involving a region of instability where (∂*P*_⊥_/∂*D*)_*N,A,T*_ > 0; see Fig. 4a for the case *σ* = 27.73 nm^−2^. This first-order phase transition becomes less pronounced as *σ* → 25.60 nm^−2^ (star in Fig. 5b) and, surprisingly, at *σ* < 25.60 nm^−2^, the transformation between the bilayer ice and the liquid becomes smooth. As an example, we include in Fig. S1 the *P*_⊥_(*D*) for the case *σ* = 24.54 nm^−2^, for which the bilayer ice-liquid transition is smooth.

One would expect that the star in Fig. 5b is a crystal-liquid critical point, analogous to the critical point found in liquid-gas phase transitions. However, we note that it is not clear how a liquid-crystal first-order phase transition should end^66^. Even in the case of bulk systems, there is no evidence of a liquid-crystal critical point. Based on the profile of the *P*_⊥_(*D*) curves at constant *σ* (Fig. 5a), our data does not seem to indicate the presence of an inflection point, i.e, a value of D for which (∂^2^*P*_⊥_/∂*D*^2^)_*N,A,T*_ = 0, which would imply the existence of a liquid-bilayer ice critical point. We also note that even across the crystal-liquid transition (at *σ* ≥ 25.60 nm^−2^), the slope of *P*_⊥_(*D*) approaching the spinodal lines is not close to zero (Figs 4a and 5a); in the case of typical liquid-gas first-order phase transitions, the compressibility diverges at the spinodal lines, i.e., implying that the slope of *P*(*V*) is indeed zero. Accordingly, we interpret the star in Fig. 5b to indicate a transition from bilayer ice-liquid first-order phase transition at *σ* ≥ 25.60 nm^−2^, to continuous bilayer ice-liquid transformation at *σ* ≤ 24.45 nm^−2^ (see dashed-line in Fig. 5b). Similar results were found in previous computer simulations of confined water^21,32,67^. One may also wonder if the *mono*layer ice-liquid phase transition in Fig. 5b at *σ* < 17.06 nm^−2^ also evolves into a continuous crystal-liquid transformation at *σ* > 17.06 nm^−2^. In our simulations, we could only detect a region of instability [(∂*P*_⊥_/∂*D*)_*N*,*A,T*_ > 0] for *σ* ≤ 17.07 nm^−2^. At higher values of *σ*, the behavior of *P*_⊥_(*D*) either shows a region of instability (associated to the monolayer ice-liquid transition) that is too small to be detected, or the instability region moves to very small values of D and hence, it disappears altogether. Accordingly, in Fig. 5b, we extend the orange and red lines at *σ* ≥ 17.07 nm^−2^ with dashed-lines.

Next, we discuss the phase diagrams of Fig. 5c and d. In both cases, we include the same phases of water and corresponding spinodal lines indicated in Fig. 5b. Briefly, the orange/red lines are the spinodal lines associated to the monolayer ice-liquid first-order phase transition; the magenta/brown lines are the spinodal lines associated to the bilayer ice-liquid first-order phase transition. The black and green lines are the spinodal lines associated to the liquid-to-vapor and vapor-to-liquid phase transitions, respectively. These phase diagrams need to be interpreted carefully since they may be confusing. For example, from Fig. 5d, the monolayer ice-liquid and liquid-gas coexistence regions overlap, suggesting that there could be a triple point where these phases coexist with one another. However, we note that this is not the case. These three phases form at different values of D (with same *P*_⊥_ and *σ*) and hence they cannot be found simultaneously between the graphene sheets (at a given D).

A very important result follows from Fig. 5c and d. Specifically, these figures provide the cavitation pressure *perpendicular* to the graphene sheet, ${P}_{\perp }^{cav}$, as a function of D (Fig. 5c) and *σ* (Fig. 5d). ${P}_{\perp }^{cav}$ is the minimum pressure that crystalline/liquid water can maintain before cavitation occurs, i.e., at which water must transform to the vapor state. Remarkably, Fig. 5c and d indicate that ${P}_{\perp }^{cav}$ is extremely sensitive to both *σ* and D. In the case of *bulk* SPCE water, *P*_*cav*_ ≈−150 MPa (*T* = 300 K) which is close to the theoretical cavitation pressure predicted by CNT (*T* = 300 K)^55,56,68^. Indeed, as shown in Fig. 5c, ${P}_{\perp }^{cav}$ seems to approach the corresponding cavitation pressure of bulk SPCE water for large values of D [${P}_{\perp }^{cav}=-150$ MPa at *T* = 300 K]. Remarkably, ${P}_{\perp }^{cav}$ can increase by more than 500 MPa as D decreases, specifically, ${P}_{\perp }^{cav}$ = 400 MPa at *D* ≈ 0.9 nm, i.e., at walls separations for which bilayer ice can form^33,38^. The wide range of values of ${P}_{\perp }^{cav}$ for nanoconfined water may have important implications in the design/performance of nanoscale systems in humid environments^7,17^.

### Water Phase Behavior at Constant Reservoir Pressure

**Figure 6:**
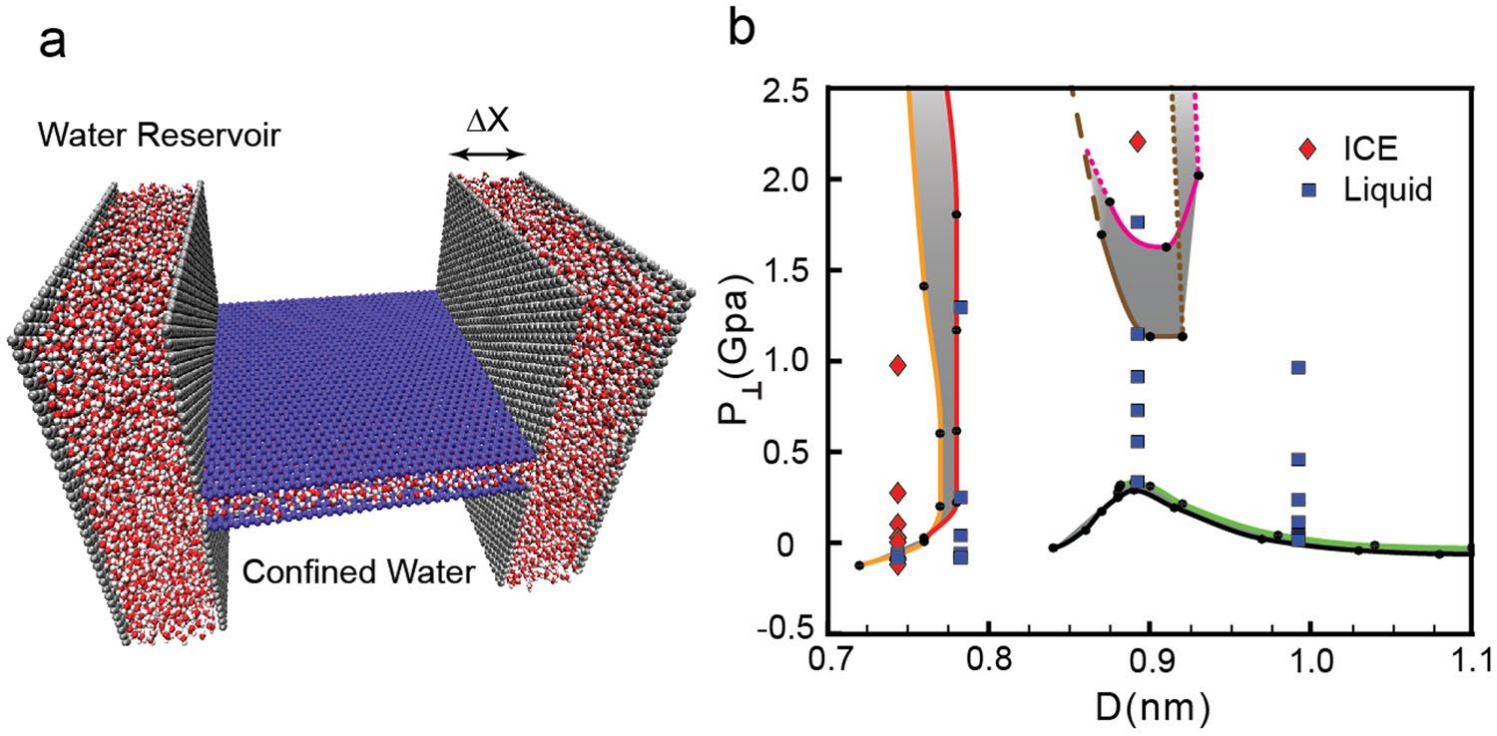
(**a**) Snapshot of the system employed to study water confined by graphene sheets at constant chemical potential, i.e., where confined water is in contact with a water reservoir. The confined system corresponds to water confined by the graphene sheets indicated in purple (separated by a distance D). The water reservoir is maintained at a target pressure *P*_*res*_ by adjusting the distance Δ*x* between the graphene sheets shown in grey. (**b**) Phase diagram of Fig. 5b, obtained using the system shown in Fig. 1a, where we include the phases of water obtained from MD simulation of the system shown in (a). As expected, MD results obtained with the systems shown in (a) and Fig. 1a are consistent with each other.

In order to test the consistency of the phase diagram in Fig. 5, we perform independent computer simulations of water confined by graphene sheets at constant A, D, and chemical potential *μ* (*T* = 300 K). Specifically, we consider the system configuration shown in Fig. 6a where water confined by two graphene sheets is in equilibrium with a water reservoir. The pressure of the reservoir, *P*_*res*_, is controlled indirectly, by fixing the reservoir’s wall-wall separation Δ*x*. The confined water is located between the graphene walls shown in purple in Fig. 6a; these sheets have a surface area *A* = 64.713 nm^2^ and are separated by a distance D. In a given simulation, we measure the force on the reservoir (grey) walls. This provides the pressure of the *reservoir*, which is the *external* pressure of the confined volume, *P*_*res*_. In addition, we also measure the pressure on the purple graphene sheets, which corresponds to *P*_⊥_. We note that the chemical potential of the system is then, identical to the chemical potential of the reservoir which can be considered to be the chemical potential of bulk water at *T* = 300 K and *P* = *P*_*res*_. Performing MD simulations using the configuration of Fig. 6a allows us to compare the phase behavior of water confined between the purple graphene walls with the phase diagram of Fig. 5. We note that setups similar to Fig. 6a have been recently used to study water confined by graphene walls at high pressure^23,69^.

To test the phase behavior of water summarized in Fig. 5, we perform MD simulations of the system shown in Fig. 6a at *D* = 0.75, 0, 79, 0.90, 1.00 nm. For each value of D, we vary Δ*x* in order to cover the values −0.2 < *P*_⊥_ < 1 GPa. The simulated (*D*, *P*_⊥_) points are indicated in Fig. 6b by red diamonds and blue squares. Red diamonds represents states where water confined between the purple graphene sheets in Fig. 6a crystallized (into monolayer or bilayer ice); blue squares represent states where water remained in the liquid state. Included in Fig. 6b is the phase diagram reported in Fig. 5c, obtained from the MD simulations of the system shown in Fig. 1a. It follows from Fig. 6b that the simulations performed by both methodologies (Figs 1a and 6a) are consistent. Specifically, the red diamonds (ice) in Fig. 6b are located mostly in the same regions where the ices are observed in the reported phase diagram of Fig. 5c, while the blue squares (liquid) in Fig. 6b are located mostly in the region corresponding to the liquid state in Fig. 5c.

The configuration of Fig. 6a can also be used to compare *P*_⊥_ and *P*_*res*_. It is well-known that for anisotropic systems consisting of water in slab geometry, these two pressure can be very different at the nanoscale^59,70^. Moreover, depending on the application, it may be more useful to have access to water’s phase behavior for a given *P*_*res*_ than at a given *P*_⊥_. Accordingly, for comparison, we include in Fig. 5b (blue lines) the path followed by the system when we maintain *P*_*res*_ at constant values, i.e. ${P}_{\perp }{(D)}_{{P}_{res}}$. Of particular interest is the behavior of the system along the path where *P*_*res*_ = 0. This situation corresponds to the system shown in Fig. 6a with the reservoir (grey) graphene sheets removed and the water reservoir remaining in contact with its vapor. Figure 5b shows that at *P*_*res*_ = 0, the system can only access the liquid phase at approximately *D* ≥ 0.92 nm and the vapor at *D* < 0.92 nm; the monolayer and bilayer ices cannot form at this conditions. By contrast, note that Fig. 5c indicates that, at *P*_⊥_ = 0, water can exist as a vapor, liquid, or monolayer ice. We also include in Fig. 5b the states sampled by the system at *P*_*res*_ = 100 and 300 MPa. At high values of *P*_*res*_, confined water can be found in the vapor, liquid, and monolayer ice.

## Discussion

We performed MD simulations of water confined by graphene sheets at *T* = 300 K over a wide range of (surface) densities *σ* and walls separations D. Our results show that, depending on D and *σ*, water confined by graphene sheets can crystallize into monolayer and bilayer square ices, or remain in the liquid and vapor states. The square ices observed in the present MD simulations are consistent with previous computational studies and experiments^23,31,52,54^ and represent crystalline forms not observed in bulk water.

The phase behavior of water confined by graphene sheets is summarized in the phase diagram of Fig. 5. That this phase diagram is very different from the phase diagram of bulk water may not be surprising. However, we note that the phase diagram of Fig. 5 is remarkably different from the phase diagram of water confined by surfaces other than graphene, such as silica-based surfaces^39^ and smooth surfaces^24,70,71^ (in the case of the silica-based surfaces, the SPCE water contact angle is *θ*_*c*_ = 108°, i,e, same as the value of *θ*_*c*_ of our graphene sheets). The underlying reason for this is the atomic-level structure of graphene and silica. While graphene is smooth at the atomic level (all C atoms in the same plane), silica^39,59^ is not. Indeed, the silica structure of the surfaces employed in these simulations^39,59^ is composed of silica tetrahedra that template the arrangement of water molecules in contact to the surfaces into hexagons. Accordingly, water confined by silica tends to form bilayer hexagonal ice, instead of square ice. In other words, surface details matter when dealing with nanoconfined water. It is this diversity of phase behaviors that makes so difficult to predict the thermodynamic states of water confined at the nanoscale.

The phase diagram shown in Fig. 5 is based on MD simulations at constant (*N*,*A*,*D*) (*T* = 300 K). We validate this phase diagram by performing independent MD simulations of water confined by graphene sheets and *in contact* with a bath reservoir (Fig. 5a). As expected, the phase behavior of water (i.e., whether it is found in the monolayer/bilayer ice, liquid, or vapor at a given D, A and N) is independent of the system considered.

The present MD simulations also provide the cavitation pressure (perpendicular to the graphene sheets) as a function of *σ* and D. We found that ${P}_{\perp }^{cav}$ is a complex non-monotonic function of *σ* and D. In particular, we found that ${P}_{\perp }^{cav}$ can be as large as 400 MPa (for *D* ≈ 0.9 nm), i.e., much larger than the cavitation pressure of bulk water at *T* = 300 K, approximately *P* = −150 MPa^55,56^. Understanding the effects of confinement on the stability of the liquid relative to the vapor is very important in technological applications^7,17^. However, most studies have focused on confined water at high pressures. Therefore, it will be of interest to study the stability of nanoconfined liquid water under tension.

We conclude by mentioning that the phase diagram of Fig. 5 is qualitatively unaffected if the water O-graphene C interactions are tuned so water contact angle with graphene is within the range 90–108°. As shown in the SI, the present results are robust relative to the specific water-graphene interactions. This is relevant since different computational models to represent the water O-graphene C interactions are available and it is not evident, a priori, how variations in water-graphene interactions may affect the results from computational studies.

## Methods

We performed extensive molecular dynamics (MD) simulations of a system composed of *N* water molecules confined between two graphene plates in slab geometry Fig. 1a. The walls are located perpendicular to the *z*-axis, at *z* = ±*D*/2 where *D* is the capillary size. Periodic boundary conditions are applied along the *x* and *y* directions and hence, the confined volume is effectively infinite along the directions parallel to the walls. Simulations are performed at constant *N*, A, D, and temperature (*N* − *A* − *D* − *T* ensemble). The system dimensions are *L*_*x*_ × *L*_*y*_ × *L*_*z*_ where *L*_*x*_ = 15.386 nm and *L*_*y*_ = 15.228 nm are the dimensions of the graphene sheets. The system is also periodic along the *z*-axis and hence, we choose *L*_*z*_ = 15.000 nm which is at least >10 times the largest value of *D* considered. This implies that there is a large space between the graphene sheets and their periodic copies along the z-axis.

We use the SPCE model for water^72^ and represent the graphene carbon (C) atoms as Lennard-Jones (LJ) particles with no partial charge. The graphene C atoms interact only with the O atoms of water and the corresponding LJ parameters are given by the Lorentz-Berthelot combination rules^73^, *σ*_*CO*_ = (*σ*_*OO*_ + *σ*_*CC*_)/2 and ${{\varepsilon }}_{CO}=\sqrt{{{\varepsilon }}_{OO}{{\varepsilon }}_{CC}}$. In these expressions, (*σ*_*OO*_ = 0.3166 nm, *ε*_*OO*_ = 0.6500 kJ/nm) are the LJ parameters of water O atoms in the SPCE model^72^, and (*σ*_*CC*_ = 0.3214 nm, *ε*_*CC*_ = 0.1510 kJ/nm) are the LJ parameters of graphene C atoms. The resulting water O-graphene C parameters are (*σ*_*CO*_ = 0.3190 nm, *ε*_*CO*_ = 0.3133 kJ/nm). Werder *et al*.^74^ showed that when these parameters are chosen, the contact angle of SPCE water in contact with graphite is *θ*_*c*_ = 107°–111°. The same water-carbon interactions parameters were used by Wang *et al*.^75^ to study water droplets in contact with graphene and graphene-based surfaces. We find that for the graphene model surface considered in this work, the contact angle of SPCE water is *θ*_*c*_ ≈ 108°.

The experimental contact angle of graphene is believed to be *θ*_*c*_ ≈ 108°^75,76^ but experimental values vary^77–82^. In addition, experiments show that graphene is ‘wettably transparent’, i.e., the contact angle of water in contact with graphene can vary considerably if a substrate is used to support the graphene sheet^83,84^. In order to explore the effects of altering the contact angle of graphene, we also perform MD simulations with modified graphene sheets for which *ε*_*CC*_ = 0.2686 kJ/nm (*ε*_*CO*_ = 0.4178 kJ/nm) while keeping *σ*_*CC*_ = 0.3214 nm. We find that the contact angle of SPCE water in contact with such a free standing ‘modified’ graphene sheet is *θ*_*c*_ ≈ 90°. As shown in Fig. S2, the phase behavior reported in Figs 2–5 is qualitatively unaffected when the original graphene model surface is replaced by the ‘modified’ graphene surface.

Our simulations are performed using the GROMACS software package^85^. The temperature is mantained constant at *T* = 300 K for all systems studied by using a Nose–Hoover thermostat (with 1-ps time constant). Electrostatic interactions are treated using a Particle Mesh Ewald (PME) solver with a reciprocal space gridding of 0.12 nm and a fourth-order polynomial interpolation. A cutoff *r*_*c*_ = 1 nm is used for the real space force calculations of the PME solver as well as for the LJ short range interactions. MD simulations are performed for 2–20 ns, depending on the diffusivity of water, and with a time step of 1 fs.

For a given number of water molecules, we first find a wall-walls separation D at which confined water is in the liquid state. Then, we increase/decrease the capillary size to explore the complete range of walls separations until the system cavitates (at large D) or crystallizes (at small D). During this process, we obtain *P*_⊥_(*D*) at constant N and identify the unstable region for the confined system.

Due to the slab confining geometry considered, and for constant (*N*, *A*, *T*), *P*_⊥_(*D*) plays the role of the pressure *P*(*V*) in a bulk liquid. As shown in the supplementary information SI, the thermodynamic condition of stability for water confined in slab geometry is

1 $${(\frac{{\rm{\partial }}{P}_{\perp }}{{\rm{\partial }}D})}_{N,A,T} < 0$$

Violation of Eq. 1 indicates the presence of a phase transition.

### Data availability

The datasets generated during and analyzed during the current study are available from the corresponding author on reasonable request.

## Electronic supplementary material

Phase Diagram of Water Confined by Graphene
