# Supplementary material for: Phase Diagram of Water Confined by Graphene
Source: Sci Rep. 2018 Apr 18;8:6228. doi: 10.1038/s41598-018-24358-3 (PMC5906694; doi:10.1038/s41598-018-24358-3)
Supplement: Supplementary file 1 — Phase Diagram of Water Confined by Graphene [file 41598_2018_24358_MOESM1_ESM.pdf]

# Supplementary Information: Phase Diagram of Water Confined by Graphene

Zhenghan Gao,[†] Nicolas Giovambattista,[*,‡,¶] and Ozgur Sahin[*,†,§]

†*Department of Physics, Columbia University, New York City, NY 10027 USA*

‡*Departments of Physics, Brooklyn College of the City University of New York, Brooklyn, NY 11210 USA*

¶*PhD Programs in Physics and Chemistry, The Graduate Center of the City University of New York, New York City, NY 10027 USA*

§*Department of Biological Sciences, Columbia University, New York City, NY 10027 USA*

E-mail: NGiovambattista@brooklyn.cuny.edu; os2246@columbia.edu

## S1. A brief thermodynamic description of a liquid confined in slab geometry

Here, we provide a brief thermodynamic description of a liquid confined in slab geometry. In particular, we show that for such a system to be thermodynamically stable, it must be that:

$$\left(\frac{\partial P_{\perp}}{\partial D}\right)_{T,N,A} < 0 \tag{1}$$

The first law of thermodynamics states that

$$\mathrm{d}U = T\mathrm{d}S - \mathrm{d}W + \mu \mathrm{d}N \tag{2}$$

where $S$ is the entropy, $\mu$ is the chemical potential, and $\mathrm{d}W$ represents the mechanical work done on the system. For a liquid confined in slab geometry (see, e.g. Refs.[1–3]), isotropic along the direction parallel to the graphene sheets, the mechanical work can be expressed as

$$\mathrm{d}W = P_{\|} D\mathrm{d}A + P_{\perp} A\mathrm{d}D \tag{3}$$

where $P_{\|}$ and $P_{\perp}$ are, respectively, the pressure along the direction parallel and perpendicular to the graphene sheets. From Eqs. 2 and 3, it follows that the Helmholtz free energy $F$ obeys

$$\mathrm{d}F = \mathrm{d}(U - TS) = -S\mathrm{d}T - P_{\|} D\ \mathrm{d}A - P_{\perp} A\ \mathrm{d}D + \mu \mathrm{d}N \tag{4}$$

If the walls surface area is constant, as it is the case in our MD simulations, then

$$\mathrm{d}F = -S\mathrm{d}T - P_{\perp}\ \mathrm{d}V + \mu \mathrm{d}N \tag{5}$$

Eq. 5 is analogous to the expression of dF for the case of a bulk liquid where $P_{\perp}$ plays the role of the bulk liquid pressure $P$. It follows that the condition of stability for a bulk liquid, $\left(\frac{\partial^2 F}{\partial^2 V}\right)_{T,N} > 0$, holds for our confined liquid ($A = \mathit{constant}$). Therefore, the condition of stability for a confined liquid in slab geometry, with constant wall surface area, is

$$\left(\frac{\partial P_{\perp}}{\partial D}\right)_{T,N,A} < 0 \tag{6}$$

## S2. Continuous bilayer ice-liquid transformation

As shown in Fig.5b of the main manuscript, the transformation between the bilayer ice and the liquid is a first-order phase transition for $\sigma > 25.60$ nm$^{-2}$ (star in Fig.5b). An example of this first-order phase transition is shown in Fig.4 of the main manuscript for $\sigma = 27.73$ nm$^{-2}$. Here, we describe briefly the case of a continuous bilayer ice-liquid transformation for the case $\sigma = 25.60$ nm$^{-2}$.

Fig.S1a shows $P_{\perp}(D)$ for $\sigma = 25.60$ nm$^{-2}$. A comparison with Fig.4a shows that at $\sigma = 25.60$ nm$^{-2}$ there is only one instability region at $D = 1.21$ nm due to the liquid-vapor phase transition, with no signature of a bilayer ice-liquid first-order phase transition. Yet, we observe that at very small D, the system crystallizes into the same bilayer ice reported at $\sigma = 27.73$ nm$^{-2}$.

Evidences of the bilayer ice-liquid transformation occurs is provided in Figs. S1(b)-(d), where we include the radial distribution function, MSD, and density profile of water at selected values of $D$. Specifically, Figs. S1b indicates that water molecules arrange into two monolayers at all graphene sheet separations. Figs. S1(c) and (d) indicate that as $D \to 0.77$ nm, the MSD decreases rapidly and becomes almost constant for $D \approx 0.77$ nm while, simultaneously, the RDF of water exhibits more pronounced extrema. In other words, for $D \approx 0.77$ nm, liquid water becomes ice. We note that the location of maxima and minima in the RDF of Fig.S1(d) for $D = 0.77$ nm and in Fig.4d of the main manuscript are identical, indicating that the same bilayer ice forms at both surface densities.

## S3. Effects of Water-graphene interactions on the Phase Behavior of Confined Water

The contact angle of water in contact with graphene is approximately $\theta_c = 96°$ in theory,[4] but experimental results vary, finding $90 \leq \theta_c \leq 108°$.[5–7] Our results are based on water

O-graphene C interactions with a Lennard Jones $\epsilon_{CO}$ parameter of $\epsilon_{CO} = 0.15104$ kJ/mol which was chosen in order to reproduce a water contact angle of $\theta_c = 108°$. A natural question is whether the phase behavior of water confined between graphene sheets reported in the main manuscript is sensitive to the specific water O-graphene C interactions chosen. In order to address this question, we perform MD simulations for the same system shown in Fig.1a of the main manuscript but where $\epsilon_{CO} = 0.26860$ kJ/mol. For this value of $\epsilon_{CO}$, we find that $\theta_c = 90°$.

In Fig.S2, we show the behavior of $P_\perp(D)$ for selected values of sigma for $\epsilon_{CO} = 0.15104$ kJ/mol ($\theta_c = 108°$, solid lines) and $\epsilon_{CO} = 0.26860$ kJ/mol ($\theta_c = 90°$, dashed lines). It follows from Fig.S2 that the phase behavior of water confined by graphene sheets is indeed robust relative to variations in water-graphene interactions with only minor changes for $90° \leq \theta_c \leq 108°$.

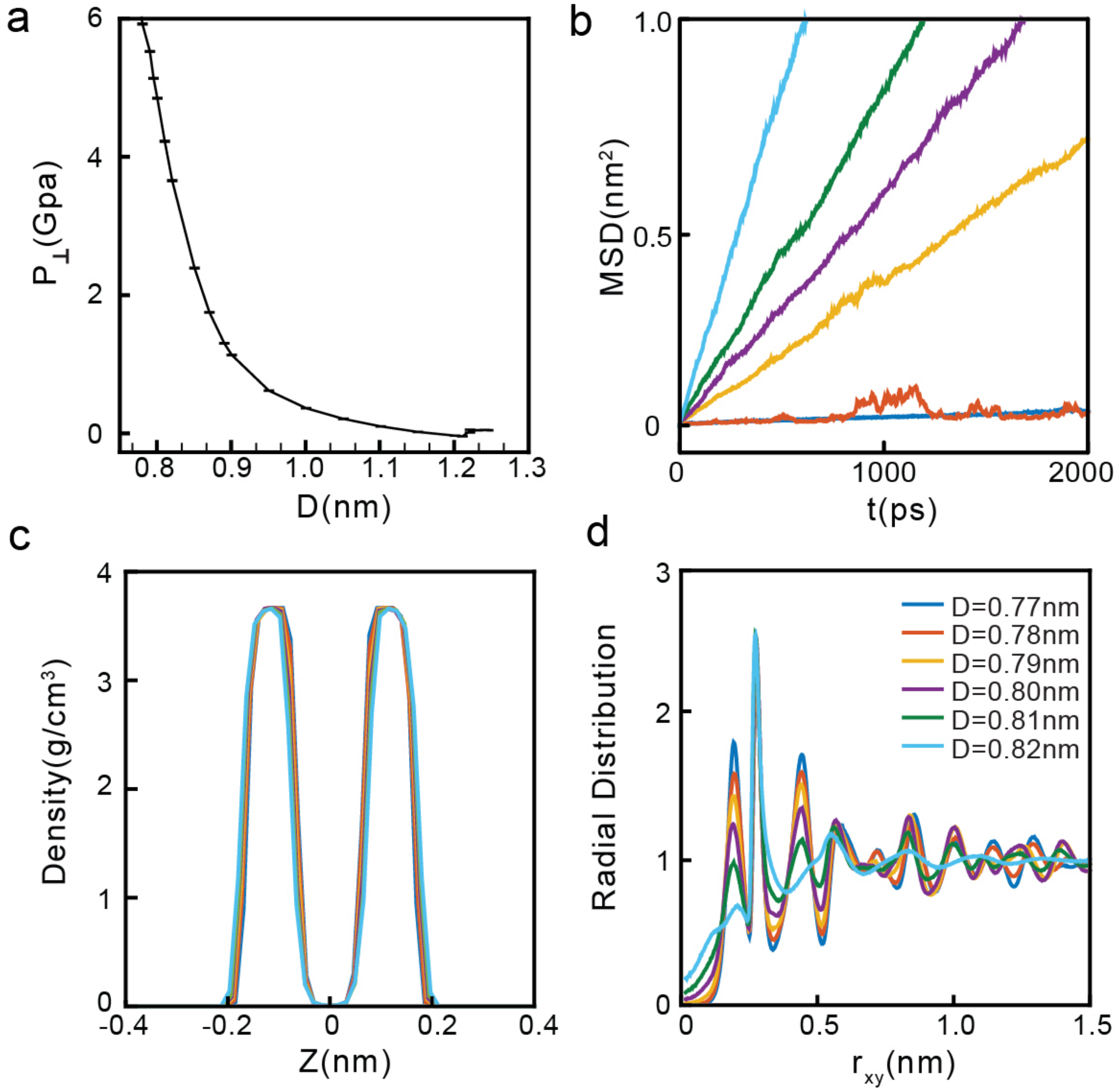


Fig.S1: *Continuous* bilayer ice-liquid transition at $\sigma = 25.60$ nm$^{-2}$. (a) $P_{\perp} - D$ curve. (b) Mean square displacement. (c) Transverse density profile along z direction. (d) Lateral Oxygen-Oxygen radial distribution.

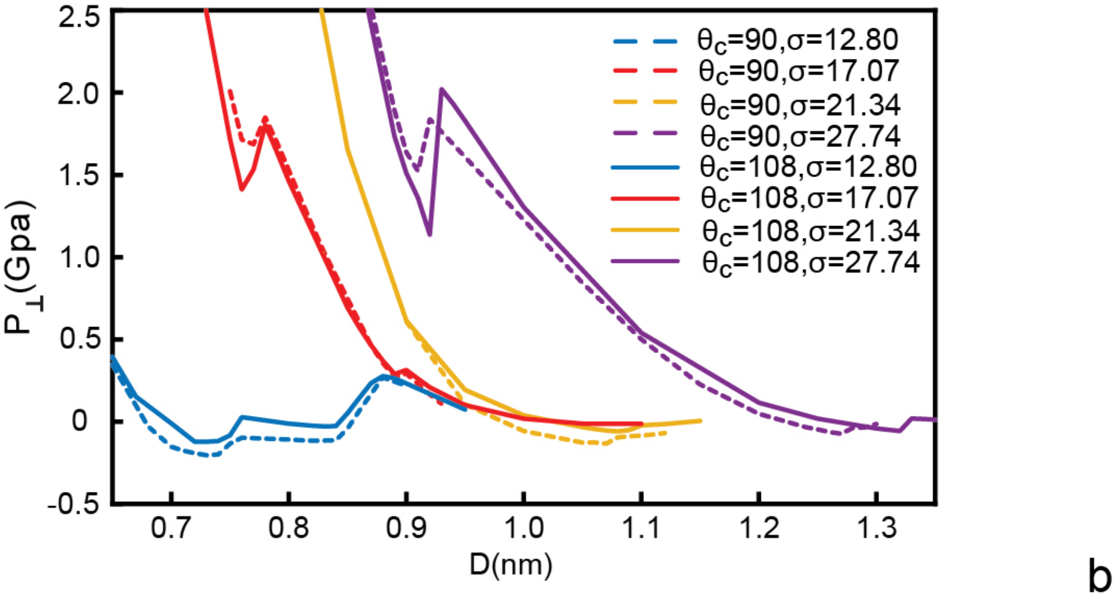


Fig.S2: Pressure perpendicular to the graphene sheets for selected surface densities $\sigma$. Solid lines are taken from Fig.1a of the main manuscript and correspond to water O-graphene C interactions with Lennard-Jones parameter $\epsilon_{CO} = 0.15104$ kJ/mol ($\theta_c = 108°$). Dased lines correspond to $\epsilon_{CO} = 0.26860$ kJ/mol ($\theta_c = 90°$). In both cases, we find basically the same phase behavior of water indicating that our results are robust relative to variations in $\epsilon_{CO}$ ($\theta_c$).

# References


1. Klapp, S.; Schoen, M. *Reviews In Computational Chemistry*; 2007; Vol. 24.

2. Truskett, T. M.; Debenedetti, P. G.; Torquato, S. Thermodynamic implications of confinement for a waterlike fluid. *The Journal of Chemical Physics* **2001**, *114*, 2401–2418.

3. Giovambattista, N.; Rossky, P. J.; Debenedetti, P. G. Phase transitions induced by nanoconfinement in liquid water. *Physical review letters* **2009**, *102*, 050603.

4. Shih, C.-J.; Wang, Q. H.; Lin, S.; Park, K.-C.; Jin, Z.; Strano, M. S.; Blankschtein, D. Breakdown in the wetting transparency of graphene. *Physical review letters* **2012**, *109*, 176101.

5. Shin, Y. J.; Wang, Y.; Huang, H.; Kalon, G.; Wee, A. T. S.; Shen, Z.; Bhatia, C. S.; Yang, H. Surface-energy engineering of graphene. *Langmuir* **2010**, *26*, 3798–3802.

6. Wang, S.; Zhang, Y.; Abidi, N.; Cabrales, L. Wettability and surface free energy of graphene films. *Langmuir* **2009**, *25*, 11078–11081.

7. Rafiee, J.; Mi, X.; Gullapalli, H.; Thomas, A. V.; Yavari, F.; Shi, Y.; Ajayan, P. M.; Koratkar, N. A. Wetting transparency of graphene. *Nature materials* **2012**, *11*, 217–222.
